# Genomic selection in forest trees comes to life: unraveling its potential in an advanced four-generation *Eucalyptus grandis* population

**DOI:** 10.3389/fpls.2024.1462285

**Published:** 2024-10-30

**Authors:** Damián Duarte, Esteban J. Jurcic, Joaquín Dutour, Pamela V. Villalba, Carmelo Centurión, Dario Grattapaglia, Eduardo P. Cappa

**Affiliations:** ^1^ Forestal Oriental, UPM, Paysandú, Uruguay; ^2^ Instituto Nacional de Tecnología Agropecuaria (INTA), Instituto de Recursos Biológicos, Centro de Investigación en Recursos Naturales, Buenos Aires, Argentina; ^3^ Consejo Nacional de Investigaciones Científicas y Técnicas (CONICET), Buenos Aires, Argentina; ^4^ Instituto de Agrobiotecnología y Biología Molecular (IABiMo), INTA-CONICET, Buenos Aires, Argentina; ^5^ Plant Genetics Laboratory, EMBRAPA Genetic Resources and Biotechnology, Brasilia, Brazil

**Keywords:** genomic selection effectiveness, seedling stage, predicted genomic breeding value, observed breeding value, *Eucalyptus*

## Abstract

Genomic Selection (GS) in tree breeding optimizes genetic gains by leveraging genomic data to enable early selection of seedlings without phenotypic data reducing breeding cycle and increasing selection intensity. Traditional assessments of the potential of GS in forest trees have typically focused on model performance using cross-validation within the same generation but evaluating effectively realized predictive ability (RPA) across generations is crucial. This study estimated RPAs for volume growth (VOL), wood density (WD), and pulp yield (PY) across four generations breeding of *Eucalyptus grandis*. The training set spanned three generations, including 34,461 trees with three-year growth data, 6,014 trees with wood quality trait data, and 1,918 trees with 12,695 SNPs (single nucleotide polymorphisms) data. Employing single-step genomic BLUP, we compared the genomic predictions of breeding values (GEBVs) for 1,153 fourth-generation full-sib seedlings in the greenhouse with their later-collected phenotypic estimated breeding values (EBVs) at age three years. RPAs were estimated using three GS targets (individual trees, trees within families, and families), two selection criteria (single- and multiple-trait), and training populations of either all 1,918 genotyped trees or the 67 direct ancestors of the selection candidates. RPAs were higher for wood quality traits (0.33 to 0.59) compared to VOL (0.14 to 0.19) and improved for wood traits (0.42 to 0.75) but not for VOL when trained only with direct ancestors, highlighting the challenges in accurately predicting growth traits. GS was more effective at excluding bottom-ranked candidates than selecting top-ranked ones. The between-family GS approach outperformed individual-tree selection for VOL (0.11 to 0.16) and PY (0.72 to 0.75), but not for WD (0.43 vs. 0.42). Furthermore, higher levels of relatedness and lower genotype by environment (G × E) interaction between training and testing populations enhanced RPAs for VOL (0.39). In summary, despite limited effectiveness in ranking top VOL individuals, GS effectively identified low-performing individuals and families. These multi-generational findings underscore GS’s potential in tree breeding, stressing the importance of considering relatedness and G × E interaction for optimal performance.

## 1 Introduction

Genomic selection (GS) has become a transformative approach in animal and plant breeding in the last two decades (Meuwissen et al., 2001). GS harnesses genotype and phenotype data from a training (or reference) population to predict genomic breeding values of genotyped but non-phenotyped selection candidates. In contrast to traditional breeding methods, which rely exclusively on phenotypic and pedigree information, GS offers a unique advantage in long-lived perennial trees. It significantly shortens the breeding cycle by enabling early-stage assessment of genetic potential through marker-based genotype evaluation (Lebedev et al., 2020). Consequently, the need for protracted and costly field testing of offspring, typically required for phenotypic evaluation, is reduced or even eliminated. Such early assessment is especially valuable in trees since most economically relevant traits either manifest late in development or are challenging and costly to measure (Jurcic et al., 2023). Ultimately, these advancements are expected to lead to a substantial increase in genetic gain per unit of time in a tree breeding program (Grattapaglia, 2022).

The potential of GS in forest trees is frequently assessed through measures such as the prediction accuracy (correlation between genomic predictions of breeding values -GEBVs- and observed phenotypes) or predictive ability (correlation between GEBVs and true breeding values). Typically, these model performances are evaluated through cross-validation analyses on individuals within the same generation, subdivided into calibration and validation sets (see Grattapaglia, 2022 for a recent review). Cross-validation is a valuable technique for evaluating and contrasting genomic prediction models in terms of accuracy and bias (Putz et al., 2017). Nevertheless, prediction accuracies obtained from cross-validation may not accurately reflect the GS accuracy expected across multiple generations in breeding programs. Only a few empirical GS studies in forest trees have looked at predictions spanning more than two generations (Bartholomé et al., 2016; Haristoy et al., 2023).

Accurate assessments of prediction accuracy, or predictive ability, can only be made after the completion of the GS cycle (*i.e.*, *a posteriori*; Werner et al., 2020). That is, it is crucial to match the GEBVs predictions of young candidates to their phenotypic values assessed in experimental field trials. This approach confirms the accuracy of genomically selected breeding parents and offspring candidates and provides insights on the correspondence between the proportion of selection candidates selected using genomic information *versus* those that would be selected based on their measured phenotypes (Herter et al., 2019). Only through such rigorous evaluation can tree breeders assess the effectiveness of GS in tree breeding. While few studies have explored the realized predictive ability (RPA) in crops (Rutkoski et al., 2015; Herter et al., 2019), this question has received even less attention in tree breeding. In a recent study, however, the effectively RPA was evaluated for volume growth in an operational program of hybrid *Eucalyptus* (Simiqueli et al., 2023). That study compared the predictive ability using diverse training populations, with different levels of relatedness to the selection candidates. GEBVs were estimated for a set of 197 selection candidates at the seedling stage, which were grown and eventually phenotyped for volume growth at age six years. The highest RPA were achieved when GS models were trained only with the direct parents (*n* = 18) of the selection candidates, in line with earlier findings in *Pinus pinaster* (Bartholomé et al., 2016) and *Pseudotsuga menziesii* (Thistlethwaite et al., 2019).

The aim of this study was to assess the RPA for growth and wood quality traits across generations in an operational breeding program of *Eucalyptus grandis* (Hill ex Maiden). GEBVs of seedling candidates for volume growth, wood density, and pulp yield traits were matched to their estimated breeding values (EBVs) from the observed phenotypes at selection age using data from a four-generation *E. grandis* breeding population. Our training population consisted of three generations, including 34,461 trees with recorded growth traits at around age 3, 6,014 trees with recorded wood quality traits, and 1,918 genotyped trees with 12,695 SNPs obtained with the EUChip60K or the Axiom Euc72K arrays. Building on a previous report (Simiqueli et al., 2023) we also evaluated the effectiveness of using exclusively genotype and phenotype data of the 67 direct or immediate ancestors (including parents, grandmothers, and great-grandmothers) as a training population. Using single-step genomic best linear unbiased prediction (ssGBLUP) we predicted the GEBVs of 1,153 fourth-generation full-sib selection candidate seedlings. Subsequently, these trees were grown and phenotyped at age three providing the phenotypic data for estimating their EBVs.

Our main objectives were: (1) to calculate the RPA, that is the correlation between the GEBVs and the EBVs estimated from the observed phenotypes using the conventional pedigree-based selection and (2) to determine the coincidence rate between the proportions of genomically and phenotypically selected trees and families in the top and bottom tiers of the ranks. Two different training sets based on genotype and phenotype data spanning the first three generations were used: (1) including all 1,918 genotyped trees, or (2) using only the data for the 67 direct ancestors of the selection candidates. The RPAs were evaluated for three different selection targets: individual trees overall selection candidates, individual trees within families, and families. Two selection criteria were implemented: single-trait selection, where the top-ranked trees or families were chosen based on the GEBVs for each trait separately, and multiple-trait selection using a selection index for the three studied traits. To assess the efficacy of GS, we compared the genomically selected sets with a random sample of individuals or families for comparison (Resende et al., 2017; Herter et al., 2019). Finally, we further investigated the impact of variable levels of the average relationship and genotype by environment (G × E) interaction between training and testing sets on the RPA, using two genomic prediction models, ssGBLUP and the classical genomic best linear unbiased prediction (GBLUP).

## 2 Materials and methods

### 2.1 Plant material, phenotypic measurements, and trial description

This study was performed on a subset of *Eucalyptus grandis* (Hill ex Maiden) (hereafter *E. grandis*) trees of the fourth-generation breeding population belonging to UPM-Forestal Oriental S.A. In total, 35,378 trees in 13 open pollinated half-sib (HS) and full-sib (FS) family trials planted between 1992 and 2020 were measured at around age three (excepting for three second-generation trials assessed at age 13) for diameter at breast height (DBH, at 1.3 m from the ground, cm) and total tree height (HT, m), and their wood volume (VOL, m^3^) estimated. Near-Infrared (NIR) spectroscopy was used to estimate pulp yield (%, PY), and wood density (kg.m^-3^, WD) for a subset of 6,869 trees. This *E. grandis* breeding population involves four generations. The first one comprised three open-pollinated trials. The second encompassed three predominantly FS and one HS progeny trials. The third generation consisted of two HS and three FS progeny trials. Finally, the fourth generation included 54 FS families derived from 34 parents, with 1,135 trees at the seedling stage in the greenhouse. These trees were later planted in a FS progeny trial and phenotyped for growth (917 trees) and the wood quality traits mentioned (855 trees) at age three. These 54 families became 64 following pedigree correction (see below).

Each progeny trial was established using an incomplete block design (IBD) with an alpha-lattice arrangement. The trials varied between 5 to 32 replications, with 6 to 25 incomplete blocks. Trees within these blocks were planted in either single-tree or 4-tree row plots, with spacing ranging from 3.0 × 2.0 m to 4.0 × 2.25 m. Detailed information on the generation, test type, number of families, and experimental design for each of the 13 progeny trials across the four-generation *E. grandis* breeding program is provided in **Supplementary Table S1**.

Prior to the analyses, all the phenotypic data were spatially adjusted (*e.g.* Dutkowski et al., 2016) using the design effects estimated for each trait and site through a pedigree-based classical *a priori* design model. Design-adjusted phenotypic data were obtained for each tree for each trait and site by subtracting the estimated replicate, and incomplete block effects from the original phenotype. Data of all traits were standardized (mean = zero and variance = 1). The list of traits, number of trees measured for each trait, and summary statistics for all the phenotypic traits in their original scale (*i.e.*, without design-adjustment or standardization) are presented (**Table 1**).

**Table 1:** Summary of data available for the training set and selection candidates in the study.

	Trait	Populations for training set			Selection candidates	Total
**Generation**		1	2	3	4	4
**No. sites**		3	4	5	1	13
**Test type**		OP	OP-CP	OP-CP	CP	OP-CP
**No. records**	VOL	17317	9071	8073	917	35378
	WD	1045	2025	2944	855	6869
	PY	1045	2025	2944	855	6869
**Total genotyped trees**		72	817	1029	1053	2971
**Direct ancestors of selection candidates**		11	23	33	–	67
**Trait Mean (SE) All trees**	VOL	0.020 (0.000)	0.207 (0.002)	0.084 (0.001)	0.021 (0.001)	0.083 (0.001)
	WD	526.3 (1.92)	495.5 (1.11)	385.6 (0.61)	428.6 (1.06)	444.7 (0.87)
	PY	49.1 (0.09)	49.9 (0.04)	50.5 (0.04)	52.7 (0.04)	50.4 (0.03)
**Trait Mean (SE) Genotyped trees**	VOL	0.030 (0.001)	0.220 (0.005)	0.131 (0.001)	0.021 (0.001)	0.120 (0.002)
	WD	539.5 (5.52)	490.3 (1.89)	381.2 (1.02)	428.4 (1.09)	432.0 (1.15)
	PY	49.6 (0.28)	50.3 (0.06)	51.4 (0.05)	52.8 (0.04)	51.4 (0.03)

Data include the number of sites, test types, and records for volume (VOL), wood density (WD), and pulp yield (PY). Information are provided on the total number of genotyped trees, direct ancestors to the selection candidates, and mean with standard errors (SE) for each trait.

OP, Open-pollinated; CP, Control-pollinated.

### 2.2 Molecular markers

SNP marker data was obtained for a total of 2,971 trees. A subset of 1,122 trees belonging to the first, second, and third generations were genotyped with the EUChip60K Illumina chip (Silva-Junior et al., 2015), and the remaining 1,849 trees only from the third and fourth generations were genotyped with the Axiom 72K *Eucalyptus* SNP Array (ThermoFisher, Santa Clara, CA). The number of genotyped trees categorized by generation and allotted to the training sets and selection candidates are informed (**Table 1**). Analyses were conducted using the 28,177 SNP markers shared by the two genotyping platforms. SNP markers were filtered retaining those with minor allele frequency (MAF) ≥ 0.05, and Call Rate (CR) ≥ 0.95 using the R-package (www.r-project.org) synbreed (Wimmer et al., 2012). Mendelian conflicts > 0.1 were also checked using qcf90 program (Masuda et al., 2019) of the BLUPF90 family (Misztal et al., 2018). As a result, a total of 12,695 SNP markers were used in the subsequent genetic analyses.

### 2.3 Pedigree correction

Pedigree correction was done using a custom R-script and was based on the comparison of expected (pedigree) *versus* observed (molecular) additive genetic relationships (Muñoz et al., 2014) across the four generations. The pairwise additive relationship coefficients in the ***G***-matrix were examined for significant deviations from their expected values (*e.g.*, 0.25 for half-sib and 0.50 for full-sib). Subsequently, manual corrections were made, and parentage was reassigned.

For trees in the fourth generation, pedigree records of a total of 77 samples were corrected based on the SNP data. These changes primarily resulted from the identification of an unknown father (for three trees), the recognition of 23 trees with incorrect fathers, 17 trees that were not linked to any of the initially assigned parents, and the correction of 6 misidentified fathers. Additionally, 36 trees had their parentage, either father and/or mother, reassigned. Finally, the corrected pedigree showed the 1,053 trees originating from 35 parents (34 of the original parents plus 1 new unsampled father). The number of genotyped trees per family ranged from 1 to 30. Notably, the underrepresented families were generally those in which one of the parents had been corrected.

### 2.4 Statistical analysis

We performed a pedigree-based best linear unbiased prediction (ABLUP) analysis and a genomic-based ssGBLUP analysis for each trait. The ABLUP used the following individual-tree mixed model for each trait:


(1) y=Xβ+Za+e

where ***y*** is the vector of adjusted phenotypes, β is the vector of fixed effect of genetic group formed according to the degree of genetic improvement (breeding cycle generation, or introductions); ***a*** is a vector of random additive genetic effects or breeding values distributed as $a∼N\left(0,A\sigma_{a}^{2}\right)$ where ***A*** is the average numerator relationship matrix derived from the pedigree (Henderson, 1984), and $\sigma_{a}^{2}\;$ is the additive genetic variance. ***X*** and ***Z*** are the incidence matrices for the fixed and random effects; and *e* is the vector of random residuals distributed as $e∼N\left(0,I\sigma_{e}^{2}\right)$ where ***I*** is the identity matrix and $\sigma_{e}^{2}$ is the residual variance.

In order to fit the ssGBLUP models, the pedigree-based relationship ***A***-matrix of model [1] was replaced by the combined pedigree- and marker-based relationship ***H***-matrix, of the same dimension as the ***A***-matrix. Actually, only the inverse of ***H*** is needed to fit the ssGBLUP models. Therefore, the inverse of the ***H***-matrix (***H*** ^–1^) was obtained as follows (Legarra et al., 2009; Misztal et al., 2009; Aguilar et al., 2010; Christensen and Lund, 2010):


$$H^{-1}=A^{-1}+\left[\begin{array}{cc} 0 & 0\\ 0 & \lambda \left(G^{-1}-A_{22}^{-1}\right) \end{array}\right]$$

where λ scales the differences between genomic and pedigree-based information, ***G*** ^–1^ is the inverse of the genomic relationship matrix (***G***-matrix), and $A_{22}^{-1}$ is the inverse of the pedigree-based relationship matrix for the genotyped individuals. In all our analyses, the scale parameter was set to λ = 0.95.

The narrow-sense individual heritability for the ABLUP and ssGBLUP analyses, ${\hat{h}}^{2}$ , was estimated for each trait as: ${\hat{h}}^{2}={\hat{\sigma }}_{a}^{2}/\left({\hat{\sigma }}_{a}^{2}+{\hat{\sigma }}_{e}^{2}\right)$ , where ${\hat{\sigma }}_{a}^{2}$ represents the estimated genetic variance, and ${\hat{\sigma }}_{e}^{2}$ denotes the estimated residual variance from the individual-tree mixed model [1] using pedigree-based (ABLUP) and the combined pedigree- and marker-based (ssGBLUP) relationship matrices.

The blupf90+ software of the BLUPF90 family (Misztal et al., 2018) was utilized to estimate the variance components and their functions (heritabilities) and to predict the breeding values for the ABLUP and the ssGBLUP models (Equation 1).

### 2.5 Training populations

Our training population consisted of trees belonging to three generations prior to the fourth generation where the selection candidates were sampled. These three generations included 34,461 trees with recorded growth traits at around age three, 6,014 trees with recorded wood quality traits, and 1,918 of them genotyped. Following recent realized predictive ability results in *Eucalypts* (Simiqueli et al., 2023), we also evaluated an alternative training model including exclusively a set of 67 ancestors of a total of 94 in the three prior generations that had direct relationship with the fourth-generation selection candidate trees. This subset consisted of parents (33 out of 34), grandmothers (23 out of 31), and great-grandmother (11 out of 29), all of them both phenotyped and genotyped (see **Table 1**). For this reduced training population, all other 1,851 trees exhibiting more distant genetic relationships with the selection candidate were not used. The network representation of the pedigree-based relationship matrix for the 2,971 genotyped trees (**Supplementary Figure S1**) displays a central core cluster comprising the 67 direct ancestors along with relatives such as aunts (0.125) and great-aunts (0.0625) from the three prior generations, all of which have genetic connections to the 1,053 fourth-generation selection candidate trees. The outer ring cluster, which includes slightly fewer than 1,851 trees, exhibits more distant genetic connections (< 0.001) with the selection candidates, excluding, for example, aunts and great-aunts from the fourth-generation selection candidate trees.

### 2.6 Realized predictive abilities

Realized predictive abilities (RPAs) were evaluated for 825 selection candidates (out of 1,053) with genotype and phenotype data for the three traits studied, originating from 64 families following pedigree correction (originally 54) of the fourth generation. RPAs were calculated for three GS approaches that varied in terms of the individual and family selection target. In the first approach, RPAs were calculated for the top and bottom 90 (11% of 825) overall ranked selection candidates irrespective of family structure. In the second approach, the RPAs were calculated considering the 11% top and bottom-ranked trees within each one of the top 10 families based on their average GEBVs. The family average GEBVs was calculated based on the average GEBVs of their offspring members. To ensure precise calculation of the genotypic means for the families, data for 54 families (out of 64) each containing six or more individuals were utilized (Rios et al., 2021). Each family was represented by 9 trees, except one family that only had 6 trees measured for PY (*n* = 87). Finally, a third GS approach consisted in calculating the RPAs for the 10 (19% of 54) top and bottom-ranked families based on their average GEBVs.

The RPAs were assessed under two selection criteria: single-trait and multiple-trait. Under single-trait selection, trees or families were selected for each studied trait separately. In the multiple-trait selection, trees or families were selected based on a selection index (Index) combining the three traits with equal weight set at 0.33: Index = 0.33 × GEBV_VOL_ + 0.33 × GEBV_WD_ + 0.33 × GEBV_YP_, where GEBV_VOL_, GEBV_WD_, and, GEBV_YP_ are the GEBVs for the VOL, WD, and PY, respectively, from the single trait ssGBLUP analysis (Equation 1).

To further evaluate the effectiveness of GS, following previous a approach (Resende et al., 2017; Herter et al., 2019), we calculated the difference in average estimated breeding values (EBVs) between the genomically ranked top or bottom trees (or families) and a random sample of an equal number of trees (or families).

RPAs were calculated by the Pearson correlation between the GEBVs obtained from the ssGBLUP model and their estimated EBVs from the pedigree-based ABLUP analysis. RPAs were calculated for all the combinations of two training populations (all 1,918 trees in the prior three-generations, or only the 67 direct ancestors), three selection candidate targets (*i.e.*, individual-trees, individual-trees within families, and families), and two selection criteria (single- and multiple- trait). Family averages were determined by calculating the mean of the GEBVs or EBVs for the families with six or more trees.

An analysis of variance (ANOVA) on the RPAs was performed to test for differences in performance between the pedigree (ABLUP) and each one of the GS approaches and selection criteria for each trait. A Tukey’s multiple comparison test was employed at a significance level α = 0.05 to test for the significance of the difference in average breeding values of trees and families. This comparison was carried out between the different single- and multiple-trait selected samples and the corresponding random samples, for each genomic selection approach and trait.

Furthermore, we assessed the correspondence rate (%) between the number of candidate trees (or families) that would be genomically selected at the seedling stage and the number of trees (or families) that would be selected at age three years based on their EBVs for different proportions selected (5%, 11%, 15%, 20%, and 25% for individual-trees; 5%, 11%, 16%, 22%, and 27% for individual-trees within families; 9%, 19%, 28%, 37%, and 46% for families) within the top and bottom-ranked 825 individual trees and 54 families with more than six individuals.

### 2.7 Assessment of the impact of training scenarios and prediction models on the RPA for volume growth

In light of the complexity of volume growth as a predictable trait, we additionally examined some aspects affecting the observed RPAs for this critically important trait. To this end we investigated the impact of the following factors on the RPA between training and testing sets always across generations: (1) the variable levels of average additive relationships; (2) the variable levels of additive genotype by environment (G × E) interactions; and (3) different genomic prediction models, comparing ssGBLUP *versus* the classical genomic GBLUP model. Moreover, we evaluated the training population composition, including either all available genotyped trees or only the direct ancestors. To evaluate the influence of the average additive relationships, we calculated the pairwise average pedigree-based relationship between specific progeny trials used as training and testing sets (12 in total, **Table 1**). For assessing the impact of G × E interactions, we conducted an ABLUP analysis across the 12 sites with an unstructured additive genetic covariance matrix to estimate the genetic correlation between sites (Cappa et al., 2022). The estimates of the average relationships and genetic correlations between sites (G × E interactions) are summarized in **Supplementary Table S2**.

Based on these variable levels of average relationships and site correlations we built six training-to-testing scenarios across generations. These scenarios were designed to evaluate the effects of higher and lower average relationships and G × E interaction, under two analytical prediction models (ssGBLUP and GBLUP) and two training population compositions (all individuals or direct ancestors only), on the RPAs for volume growth. Specifically, the training populations included first-generation trial 2 and second-generation trials 4 and 7, while the testing population for all scenarios was the third-generation trial 8. The volume and pedigree data comprised information for 8,092, 829, and 6,967 trees from trials 2, 4, and 7, respectively, while trial 8 included 3,158 trees. The genotypic data comprised 71, 40, and 773 genotyped trees from trials 2, 4, and 7, respectively. In Trial 7, 73 out of 773 genotyped trees have direct relationships (mothers) to testing trial 8, whereas in Trial 2, 32 out of 71 genotyped trees are grandmothers of trees in trial 8. In trial 4, 8 out of 40 genotyped trees are cousins of the testing trees in trial 8. The testing trial 8 comprised 549 genotyped trees. The closest relationships between trials 7, 2, and 4 with the testing trees in trial 8 are as follows: trial 7 includes most of the mothers (85 out of 126), with the remaining mothers coming from another trial (10) and the base population (31). Trial 2 comprises most of the grandmothers (49 out of 92) of the candidate trees in trial 8, with the rest coming from the base population. Finally, trial 4 includes 61 cousins of trees in trial 8.

We employed the ABLUP to estimate the EBVs and the ssGBLUP and classical GBLUP models to estimate the GEBVs. The models were evaluated for their ability to predict phenotypic outcomes (RPAs) in the testing trial 8. The ABLUP and ssGBLUP models were fitted using Equation 1. For the classical GBLUP model, the pedigree-based matrix *A* was substituted with the marker-based matrix ***G***, but only using the subset of genotyped trees.

## 3 Results

### 3.1 Heritability estimates

Pedigree-based ABLUP heritability estimates ranged from 0.25 to 0.38, while genomic-based ssGBLUP analysis yielded slightly higher estimates ranging from 0.31 to 0.40. Wood density (WD) showed the highest heritability at 0.38 and 0.40, closely followed by pulp yield (PY) at 0.37 and 0.40 (**Table 2**), while volume growth (VOL) showed the lowest at 0.25 and 0.31 respectively for ABLUP and ssGBLUP.

**Table 2:** Additive variance ( $\sigma_{a}^{2}$ ), residual variance ( $\sigma_{e}^{2}$ ) and heritability (*h* ^2^) estimates (and approximate standard errors) obtained with the ABLUP and ssGBLUP models.

Model	Volume (VOL)	Wood Density (WD)	Pulp Yield (PY)
ABLUP			
$\sigma_{a}^{2}$	0.21 (0.01)	0.33 (0.03)	0.34 (0.03)
$\sigma_{e}^{2}$	0.62 (0.01)	0.54 (0.02)	0.57 (0.03)
*h* ^2^	0.25 (0.02)	0.38 (0.03)	0.37 (0.03)
ssGBLUP			
$\sigma_{a}^{2}$	0.25 (0.01)	0.35 (0.03)	0.37 (0.03)
$\sigma_{e}^{2}$	0.56 (0.01)	0.52 (0.02)	0.55 (0.02)
*h* ^2^	0.31 (0.02)	0.40 (0.03)	0.40 (0.03)

The values are derived from all available phenotypic information for each studied trait: Volume, Wood Density, and Pulp Yield. Abbreviation used for the models are described in the text.

### 3.2 Impact of training composition on realized predictive abilities

Realized predictive abilities (RPAs) reached considerably different values depending on the trait evaluated and the training population used, but only slightly different whether the selection target was the individual-tree or the family (**Table 3**). Low RPAs were observed for volume growth (0.11 to 0.19) irrespective of training population, with a modest improvement of 35-45% when families instead of individual trees were selected. RPAs for wood density (0.33 to 0.43) and for pulp yield (0.55 to 0.75) were substantially higher than for volume growth. For these wood properties traits, when GS models were trained only with the 67 direct ancestors, the RPA increased by 20-31% but only a slight increase of 4-7% was seen when selecting families instead of individual trees. These results are consistent with the higher heritability of wood properties traits and highlight the crucial importance of establishing a higher degree of genetic relatedness and minimizing genotype by environment interactions between the training set and selection candidates to enhance prediction abilities. Based on these results, the follow up analyses described below were carried out based exclusively on the most efficient training population composed by the 67 direct ancestors of the selection candidates.

**Table 3:** Realized predictive abilities (RPAs) estimated by a Pearson correlation between genomic estimated breeding values (GEBVs) from ssGBLUP and estimated breeding values (EBVs) from the pedigree-based ABLUP phenotypic model for the two alternative training populations: (1) 1,918 trees from the three prior generations to the selection candidates, and (2) the 67 direct ancestors to the selection candidates.

Trait	Training population with all 1,918 trees of prior generations		Training population with 67 direct ancestors	
	Individual-tree	Between-family	Individual-tree	Between-family
**Volume**	0.14	0.19	0.11	0.16
**Wood Density**	0.33	0.35	0.43	0.42
**Pulp Yield**	0.55	0.59	0.72	0.75

Results are presented for individual-tree and between-family genomic selection.

### 3.3 Efficiency of genomic selection across individual and family ranks

To evaluate the efficiency of genomic selection (GS) for the different traits, selection targets and selection criteria, the average GEBVs were calculated for the trees or families selected by the different GS approaches tested. In the first assessment the comparison was against an equivalent number of trees or families selected at random (**Table 4**). When the entire selection candidate population was considered, the average GEBVs was 0.22 for VOL, 0.30 for WD and 0.46 for PY. In the individual-tree GS approach and focusing on the top 11% (*n* = 90) genomically ranked trees, significantly higher average GEBVs were observed for all three traits compared to the random sample of candidates. Specifically, the average GEBVs was 0.70 for VOL, 0.82 for WD and 0.79 for PY compared to 0.44, 0.30 and 0.20 for the corresponding random samples. The average GEBV were slightly lower when GS was practiced based on a selection index (0.52 for VOL, 0.62 for WD, and 0.71 for PY). A similar result was observed for the second selection approach when the average GEBVs of the 11% top and bottom-ranked trees within each one of the top 10 families were considered. For the third GS approach, the family average GEBVs for the 19% (*n* = 10) top-ranked families, were also higher than the average GEBVs for an equivalent set of random families, but the differences were slightly smaller than those observed for individual-tree selection. Overall, these results show that the implementation of GS with any of the three selection approaches would result in considerably higher efficiency compared to a random sampling of an equivalent number of individual trees or families.

**Table 4:** Average and ranges of genomic estimated breeding values (GEBVs) for different genomic selection targets and variable selection criteria for the three evaluated traits.

Genomic selection target	Genomic Selection approach	VOL	WD	PY
**All selection candidates**		0.22 (-0.67 – 1.17)	0.30 (-0.39 – 0.95)	0.46 (0.17 – 0.83)
**Individual-tree GS**	**11% top-ranked (*n* = 90)**	0.70 (0.63 – 0.83)	0.82 (0.72 – 0.95)	0.79 (0.63 – 1.17)
	**11% bottom-ranked (*n* = 90)**	0.26 (0.17 – 0.30)	-0.20 (-0.39 – -0.02)	-0.40 (-0.67 – -0.28)
	**Multiple-trait index selected**	0.52 (0.26 – 0.83)	0.62 (0.34 – 0.88)	0.71 (0.07 – 1.17)
	**Equal size random sample**	0.44 (0.20 – 0.75)	0.30 (-0.37 – 0.84)	0.20 (-0.56 – 1.13)
**Individual-tree within-family GS**	**11% top-ranked (*n* = 90)**	0.67 (0.55 – 0.83)	0.77 (0.53 – 0.95)	0.71 (0.46 – 1.17)
	**11% bottom-ranked (*n* = 90)**	0.26 (0.17 – 0.32)	-0.12 (-0.39 – 0.12)	-0.36 (-0.67 – -0.20)
	**Multiple-trait index selected**	0.53 (0.31 – 0.83)	0.58 (0.34 – 0.88)	0.63 (0.07 – 1.17)
	**Equal size random sample**	0.45 (0.20 – 0.72)	0.36 (-0.33 – 0.94)	-0.06 (-0.56 – 0.50)
**Between-family GS**	**19% top-ranked (*n* = 10)**	0.66 (0.58 – 0.75)	0.75 (0.59 – 0.86)	0.67 (0.57 – 1.06)
	**19% bottom-ranked (*n* =10)**	0.29 (0.23 – 0.34)	-0.08 (-0.28 – 0.10)	-0.34 (-0.53 – -0.21)
	**Multiple-trait index selected**	0.51 (0.31 – 0.75)	0.55 (0.34 – 0.81)	0.60 (0.13 – 1.06)
	**Equal size random sample**	0.43 (0.28 – 0.68)	0.45 (0.18 – 0.81)	0.34 (-0.31 – 1.06)

See text for details.

**Figure 1:**
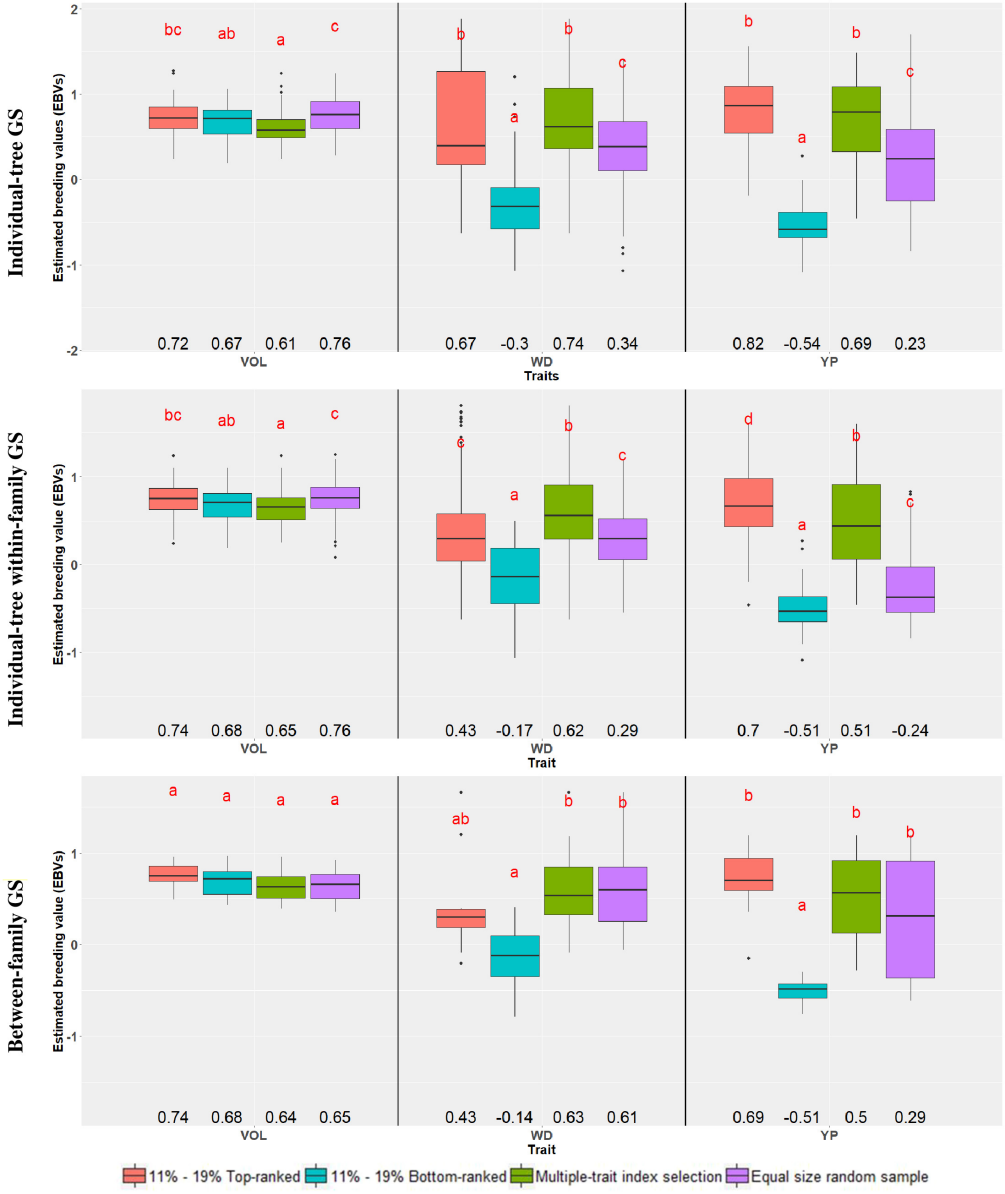
Box-plots showing the distribution of the estimated breeding values (EBVs) for each trait, across genomic selection targets and selection criteria. Within each trait, common letters above box-plots indicate nonsignificant differences (α = 0.05) according to a Tukey test. Average EBVs are indicated below the box-plots. The percentages 11% and 19% correspond to the top- and bottom-ranked trees and families, respectively. Traits are: VOL, Volume; WD, Wood Density; PY, Pulp Yield. See text for details on the genomic selection targets and selection criteria.

A second way to assess the efficiency of GS was carried out by comparing the average EBVs of the different samples of the top and bottom 11% (*n* = 90) and 19% (*n* = 10) genomically ranked trees and families, respectively, to an equivalent number of trees or families selected at random. In this case, however, a test for significant difference was applied on the average EBVs (**Figure 1**; **Supplementary Table S3**). For VOL, no significant difference was seen in the average EBVs between the top and bottom-ranked trees and families in any of the three GS approaches. The only significant difference in average EBV was detected when comparing the bottom-ranked individual trees (0.67) with the random ones (0.76) either overall or within-family. For WD, a large and significant difference in the average EBV was seen between the top and bottom-ranked individual trees both in the overall (0.67 vs. -0.30) and within-family (0.43 vs. -0.17) individual-tree selection approaches. When compared to the random sample, only the top-ranked trees selected by the overall individual-tree selection approach had a significantly higher average EBV (0.67 vs. 0.34), but the genomically bottom-ranked trees had a significantly lower average EBV by all three GS approaches (-0.30, -0.17 and -0.14 vs. 0.34, 0.29 and 0.61, respectively). For PY, a large and significant difference in the average EBV was seen between the top and bottom-ranked individual trees (or families) in all three genomic selection approaches. Similar to WD, when compared to the random sample only the top-ranked trees for PY selected by the overall individual-tree selection approach had a significantly higher average EBV, but the bottom genomically ranked trees had a significantly lower average EBV by all three GS approaches (**Figure 1**; **Supplementary Table S3**). These findings substantiate the previous results that different efficiencies are expected depending on the trait, but that GS may prove more effective in identifying candidate trees or families with inferior performance rather than those with superior performance.

**Figure 2:**
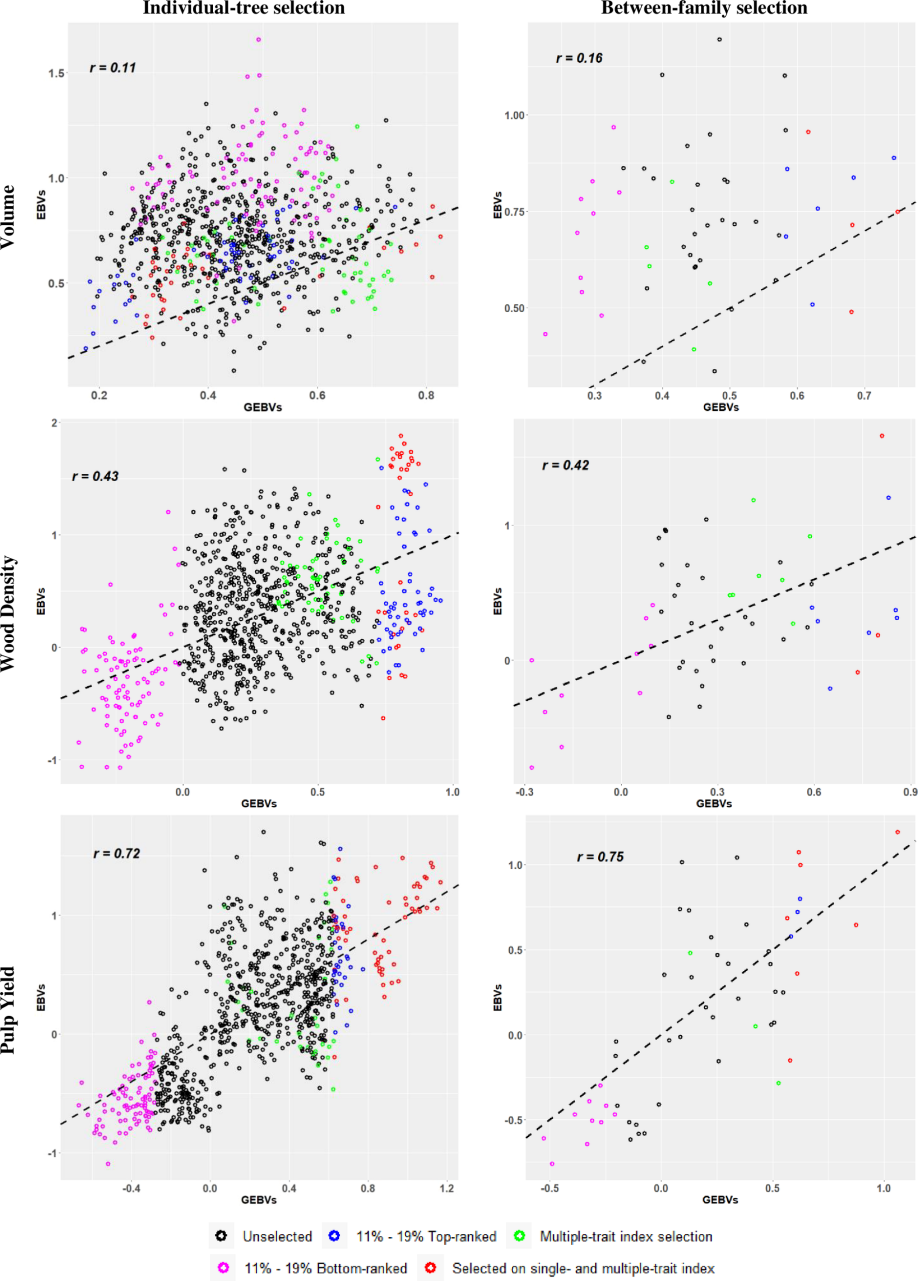
Scatter plots of the relationship between genomic estimated breeding values (GEBVs) (*x*-axis) and estimated breeding values (EBVs) from phenotypic records (*y*-axis) for individual-tree (left panels) and between-family (right panels) genomic selection approaches. Trees selected by both selection criteria (single- and multiple-trait) were identified in red. The percentages 11% and 19% correspond to the top- and bottom-ranked trees and families, respectively. Pearson´s correlation (*r*) for the plots are provided. The dashed black line acts as a reference line with intercept 0 and slope 1. See text for details.

A third way to illustrate the results of our experiment was by plotting the estimated GEBVs (*x*-axis) and corresponding EBVs (*y*-axis) for all 825 selection candidate trees and indicating the selected trees in the different target samples by different colour codes (**Figure 2**). The graphs illustrate the previously mentioned results showing that the highest RPA when overall individual-tree selection was applied was at 0.72 for PY, followed by 0.43 for WD, and 0.11 for VOL (**Table 3**). When family selection was applied, RPAs for PY and VOL improved slightly to 0.75 and 0.16, while it remained nearly constant for WD at 0.42. The colour codes also indicate the 11 - 19% top (in blue) and bottom (in pink) genomically ranked individuals and families. In red the individual trees or families that were selected based both on single-trait and multiple-trait index, and in green those that were selected only by multiple-trait index selection. These plots corroborate visually that genomic selection was essentially inefficient for VOL when compared with the results seen for for WD and PY. A large number of top-ranked trees and families for VOL by EBVs were missed by GS, while GS was efficient in identifying several trees and families in the top ranks of WD and PY. Furthermore, the plots show that GS was more efficient in identifying the bottom EBV ranked (in pink) than the top EBV ranked (in blue and red) individuals and families. As expected, this was particularly true for WD and PY but less so for VOL.

**Figure 3:**
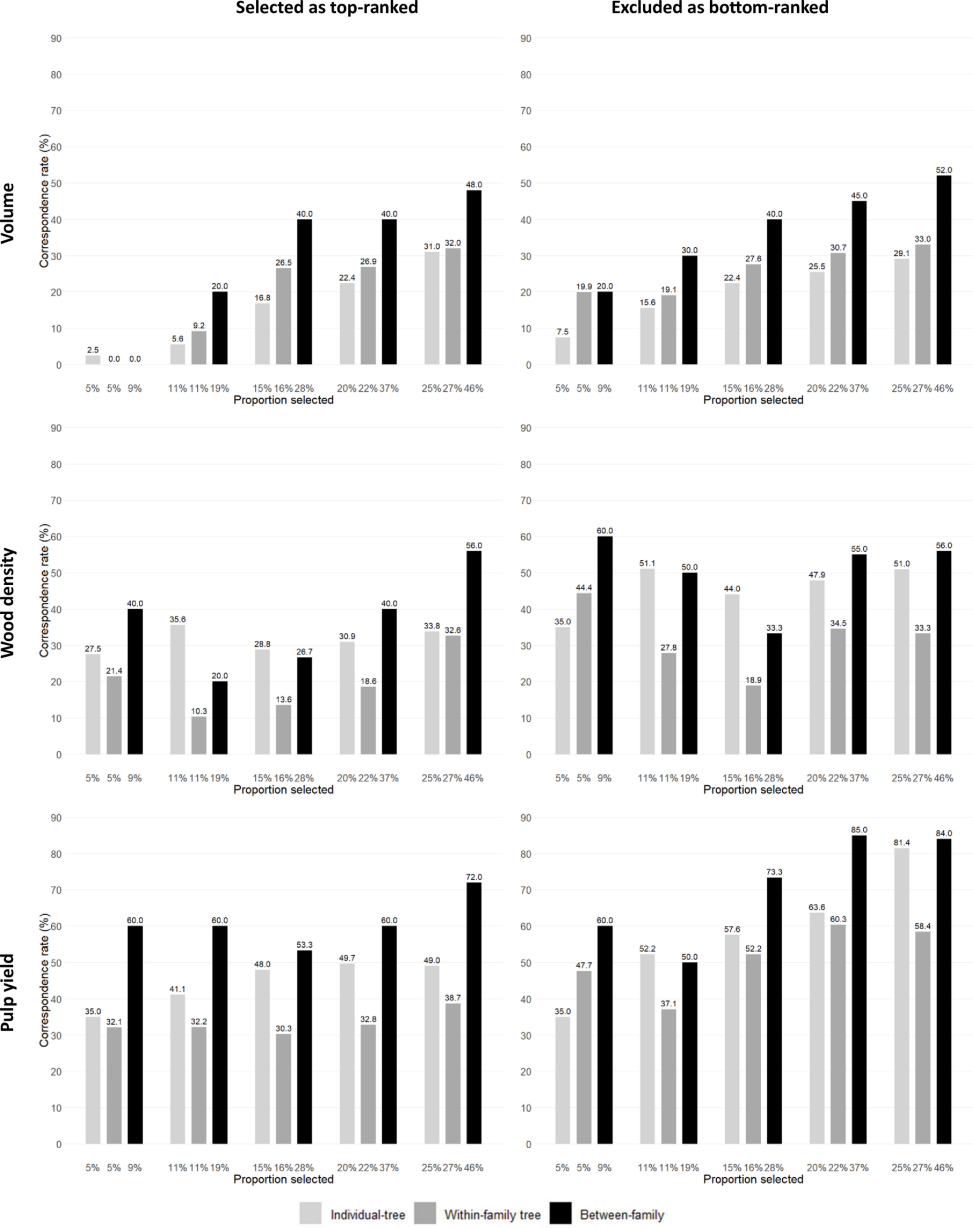
Correspondence rates (*y*-axis) between the number of trees and families that would be genomically (GEBV) and phenotypically (EBV) selected in the top or excluded from the bottom-ranked 11% individual trees (*n* = 90) and 19% families (*n* = 10) for increasing selected proportions (*x*-axis) within three different genomic selection targets (individual-tree; within-family individual tree and between-family).

A fourth way to evaluate the efficiency of GS was carried out by investigating the correspondence rate between the number of trees and families that would be genomically selected at the seedling stage and the number of trees or families that would be selected at age three years based on their EBVs for progressively increasing selected proportions (*i.e.*, progressively decreasing selection intensities). As expected, irrespective of the GS approach adopted, as the proportions of selected trees or families increased (*i.e.*, decreasing selection intensity) the correspondence rate improved (**Figure 3**; **Supplementary Tables S4**, **S5**). When examining the top-ranked trees, at the highest selection intensities (5% and 11% proportions selected) GS could only identify 2.5% and 5.6% of the top EBV ranked trees for VOL, but would satisfactorily identify 27.5% and 35.6% of the top EBV ranked trees for WD and 35% and 41.1% of the top EBV ranked trees for PY. When applying a higher selected proportion of 25%, correspondence rate improved: 31% of the top EBV ranked trees for VOL, 33.8% for WD and 49% for PY would be genomically selected. The results show that the correspondence rate would vary in efficiency when applying individual-tree GS within families when compared to individual-tree depending on the selected proportion and trait. This can be seen by the smaller correspondence rates for individual-tree GS within families (dark grey bars) when compared to the individual-tree GS rates (light grey bars) for WD and PY but not for VOL. Family selection (black bars) was generally more efficient than individual-tree selection at all proportions selected. For PY and WD, for example, GS identified 60% of the top EBV ranked families even at the highest selection intensity (9% selected proportion), indicating a strong performance of GS to identify top performing families for these late expressing traits. When examining the bottom-ranked trees or families, GS showed a generally higher efficiency in identifying the bottom-ranked trees for all three traits, confirming prior results indicating that GS was considerably more efficient for removing low performing trees and families in this particular experiment (**Figure 3**; **Supplementary Tables S4**, **S5**). Nevertheless, focusing, for example, on the results observed for PY, at the highest selection intensity of 5%, GS would identify 35% of the top EBV ranked trees and allow removing 35% of the bottom-ranked trees. At the 11% proportion selected, GS would identify 41.1% of the top-ranked trees and discard 52.2% of the lowest performers.

### 3.4 Improving realized predictive ability for volume growth

The realized predictive abilities (RPAs) for volume growth varied across the six training-to-testing-across generations scenarios studied based on variable levels of average relationships and site correlations (**Table 5**). As expected, scenarios 1 and 2 based on the lowest levels of genetic relationship between training and testing sets exhibited the lowest RPAs (0.03 and -0.12, respectively). In contrast, scenarios 3, 4, 5, and 6, based the highest training-to-testing relationship and the lowest training-to-testing G × E interactions, resulted in the highest RPAs for volume growth (0.29, 0.39, 0.24, and 0.31, respectively). These estimates correspond to substantial improvements of a minimum of 27% up to 250% in comparison with the RPAs (0.11 to 0.19) obtained when relationship and G × E were not taken into account. The highest RPA (0.39) in scenario 4, despite moderate heritability (0.26), underscores the significant role of a larger and more diverse training populations, enhanced by the ssGBLUP approach, and the inclusion of directly genotyped ancestor trees, in improving the alignment between EBVs and GEBVs. This highlights the critical influence of training dataset characteristics on the RPA across generations, particularly for complex growth traits. In terms of genomic models utilized, the ssGBLUP model exhibited superior RPAs when compared to GBLUP. Specifically, the RPAs were 0.29 versus 0.24 (a 20% increase) in scenarios 3 and 5, and 0.39 versus 0.31 (a 24% increase) in scenarios 4 and 6, respectively.

**Table 5:** Estimates of the realized predictive ability (RPA) of volume growth for the different training-to-testing scenarios studied across generations to assess the effect of the variable levels of average relationships (RELA), and genotype by environment (G × E) interaction (*i.e.*, additive genetic correlation) between training (TR) and testing (TE) populations, using two prediction genomic models (ssGBLUP and GBLUP).

Scenario	Training trial	Generation	No. phenotypic records in TR	No. genotyped trees in TR	No. phenotypic records in TE	No. genotyped trees in TE	*h* ^2^	RELA^1^	G × E	Model	RPA
1	2	1	8092	71	3158	549	0.25	0.91	0.50	ssGBLUP	0.03
2	4	2	829	40	3158	549	0.20	0.34	0.80	ssGBLUP	-0.12
3	7	2	6964	773	3158	549	0.45	2.05	0.84	ssGBLUP	0.29
4	7	2	6264^2^	73	3158	549	0.26	2.05	0.84	ssGBLUP	0.39
5	7	2	773	773	549	549	0.22	2.05	0.84	GBLUP	0.24
6	7	2	73	73	549	549	0.37	2.05	0.84	GBLUP	0.31

Trial 8 in the third generation was used as the testing population in all scenarios. Heritability (*h* ^2^) estimates were calculated using the ssGBLUP or GBLUP models. Abbreviation used for the models are described in the text.

^1^RELA values were multiplied by 10,000 for easier readability and interpretation.

^2^This number of trees (6264) differs from the total number of trees in trial 7 (6964) because 700 genotyped trees that are not related to offspring in trial 8 were excluded.

## 4 Discussion

The evaluation of genomic selection (GS) effectiveness in forest trees has primarily relied on cross-validation analyses to assess the performance of GBLUP or ssGBLUP models. This approach involves dividing the same population in the same generation into training and validation or testing sets for the evaluation of genomic prediction models. Although it provides some hints on what to expect from GS in a particular setting, this approach is far removed from the reality of operational multiple generation tree breeding. Our study therefore expands the critical assessment of the realized predictive ability (RPA) (Simiqueli et al., 2023) across multiple generations, using operational trial data of volume growth, wood density, and pulp yield collected in an active four-generation breeding program of *E. grandis*. Predictions were generated using distinct compositions of training populations sampled in three breeding generations preceding a set of 825 selection candidates, aiming at different GS targets, using different models and selection criteria. We show that the variable levels of the average relationships and genotype by environment (G × E) interaction between training and testing populations across generations, have a profound impact on the effectiveness of GS for volume growth. These insights are vital for forest breeding programs, offering a more realistic perspective on the utility and reliability of GS in multigenerational contexts.

### 4.1 Realized predictive abilities improve when training with the direct ancestors of the selection candidates

RPAs for both wood quality traits exhibited substantial improvement, increasing by 31% and 20% at the individual and family levels respectively (**Table 3**), when models were trained exclusively with the direct ancestors (parents, grandmothers, and great-grandmothers) (*n* = 67) rather than utilizing the broader set of all available genotyped trees spanning the first three generations (*n* = 1,918). These findings are in line with the outcome reported by Simiqueli et al. (2023) in *Eucalyptus*, who suggest that a possible explanation for these higher accuracies is that a focused genotyping effort targeting a training set closely related to the selection candidates might be sufficient for implementing genomic selection in forest trees. These results are also consistent with earlier results in other trees species. In *Pinus pinaster*, Bartholomé et al. (2016) demonstrated that a prediction model calibrated exclusively the 108 grandparents and parents (generations G0 and G1) of G2 candidate trees reached prediction accuracies ranging from 0.70 to 0.85 depending on the trait, equivalent to accuracies calculated by including G2 trees for a larger G0/G1/G2 calibration set with 567 trees. In *Pseudotsuga menziesii*, Thistlethwaite et al. (2019) also showed that higher prediction accuracies for juvenile height can be achieved by including only the 132 parents in the training population, rather than all 1,321 genotyped G1 trees. As proposed by Simiqueli et al. (2023), the outcomes presented in our study, along with those reported in related research, can be attributed to the longer extensions of shared haplotypes arising from direct relatedness. Early simulation studies had indicated that family relationships between selection candidates and individuals in the reference population lead to longer accumulated length of shared haplotypes which are more important than individual length of shared haplotypes in driving higher reliabilities of genomic prediction (Wientjes et al., 2013).

Utilizing both simulated data and empirical datasets, several studies on forest trees (reviewed in Grattapaglia et al., 2018; Isik, 2022) have consistently demonstrated that a high level of genetic relatedness between the calibration and validation populations, along with enlarging the size of the training set, enhanced the predictive ability. Results from (Bartholomé et al., 2016; Thistlethwaite et al., 2019; Simiqueli et al., 2023), and our own results, suggest that higher accuracy of GEBVs are driven mainly by the level of genetic relatedness between the training population and the selection candidates than by the relative size of the training population. In a recent revision of 26 published GS studies in forest trees, Isik (2022) highlighted the significant impact of relatedness (full-sibs *versus* half-sibs family structure) on the prediction accuracy, while the size of the training population (and marker number) was found to be non-significant. The author suggested that tree breeders should strategically design their training populations with a strong emphasis on genetic relatedness to those trees in the targeted selection population.

As expected, in our experiment the highest level of relatedness between the training and testing populations was found when exclusively the 67 direct ancestors (parents, grandmothers, great-grandmothers) were included in training. From the pedigree perspective, the genetic contribution of a parent to an offspring is one half (0.5), each grandparental contribution is one quarter (0.25), and each great-grandparental contribution is one eighth (0.125). The remaining 1,851 trees that could be included in the training population would make significantly smaller genetic contributions (0.0078125, 0.015625, 0.0625, and 0.125) to the selection candidates, many of which are in the outer ring cluster shown in **Supplementary Figure S1**. Including this large number of more distantly related individuals in training would considerably dilute the final genetic relationship to the selection candidates. The theoretical average relatedness between the 67 direct ancestor trees and the 825 selection candidates is one order of magnitude higher (0.022, ranging from 0.000 to 0.027) than the relatedness of all 1,918 trees (0.0038, ranging from 0.001 to 0.005) (**Supplementary Figure S2**). Our results indicate that this has a relevant positive impact on RPA despite the 29 times smaller (67 vs. 1,918) training population size. These findings once again underscore the much higher relative importance of guaranteeing close relationships between training and testing sets than using large training populations to achieve high predictive abilities. However, further investigations in forest trees are warranted to better understand and quantify the relative and simultaneous impact of relationship and size of the training population on genomic prediction accuracy.

We obtained considerably higher RPAs for WD and PY (3.9 and 6.5 times higher, respectively) compared to VOL. Further improvement was seen when a GS model was trained only with the direct ancestors (**Table 3**; **Figure 2**). Contrary to wood quality traits, however, RPA for volume growth was ~20% higher when the entire training population was used instead of only the direct ancestors. This might be due to the particular trait architecture of volume growth that makes the predictive ability more dependent on the genome-wide linkage disequilibrium (LD) than family relationships. In fact, previous studies have indicated that small reference populations result in a higher effect of family relationships on reliability of genomic predictions compared to LD, and larger reference populations result in a higher effect of LD (Clark et al., 2012; Wientjes et al., 2013). Volume growth in forest trees is known for its complex genetic architecture and lower heritability involving numerous genes and physiological processes (Cappa et al., 2013), presenting a greater challenge for prediction compared to the typically more heritable wood quality traits (**Table 2**) often associated with specific biosynthetic pathways. This result aligns with previous findings in *Eucalyptus* (Resende et al., 2017; Tan et al., 2018; Paludeto et al., 2021) and other forest trees species (Chen et al., 2018; Beaulieu et al., 2020; Lenz et al., 2020; Calleja-Rodriguez et al., 2021), where higher predictive abilities were reported for wood properties compared to growth traits.

### 4.2 Genomic selection was more effective in removing inferior individuals and rank families

Besides calculating the overall RPA across generation, ultimately the effectiveness of GS was evaluated by estimating the correspondence rates between the numbers of individuals or families that would be genomically and phenotypically selected at the top of the distribution for retention or at the bottom for exclusion. In our experimental conditions, GS was slightly more effective in identifying candidate trees or families for exclusion due to their worst performance compared to those with superior performance. This was particularly evident at the lower proportions selected (higher selection intensities) and was more pronounced for VOL than for WD and PY (**Figure 3**). At the highest selection intensities, the correspondence rates for WD and PY were in the 35 to 50% range, but for VOL they did not exceed 10 to 15%. While GS is usually seen as a way to identify top-ranked individuals, it can be useful to optimize resource allocation by excluding the bottom-performing families or trees within families during the early stages in the greenhouse, ultimately enhancing the efficiency of breeding programs.

At the top of the rank distribution and for the smallest proportions selected usually adopted in operational breeding, the correspondence rates for WD and PY were satisfactory, identifying between 35 and 41% of the top-ranked individuals. This result once again corroborates the value of GS for early selection of individual trees for these late expressing traits in eucalypts. For VOL, however, results were not as good. Only 5.6% of the top 11% highest volume growth trees were correctly predicted using GEBVs, and slightly higher proportions of 32% were only reached at much more relaxed selection intensities (**Figure 3**). Notwithstanding the intrinsic complexity of volume growth as a target trait for selection, the disparity in RPAs seen for VOL when compared to WD and PY, highlights the need for further refinement of the training populations and exploration of predictive models for this trait.

The variation in the matching proportions of individuals and families selected by GEBV and EBV can be attributed to a key difference in prediction models (ABLUP vs. ssGBLUP). As noted by Aguilar et al. (2010) (see Appendix B, Equation [B2]), the breeding value for a given tree under the genomic-based ssGBLUP model (or any genomic models fitted using ***G***-matrix) incorporates genomic information from all genotyped individuals in the reference and validation populations, including those with low or high genetic merit when predicting GEBVs. This contrasts with the pedigree-based ABLUP model used for observed EBVs, in which the breeding value for a given tree relies solely on the average performance of their parents selected in the previous generation. This key distinction can lead to ssGBLUP predicting GEBVs that are either higher or lower than observed EBVs, depending on the distribution of genetic merit within the reference and validation populations. This, in turn, could partly explain the observed variation in the efficiency of selecting and ranking superior trees or families when comparing GS using the ssGBLUP approach with phenotypic selection using an ABLUP model. Furthermore, this same distinction could also contribute to the improvement seen in RPA when predicting GEBVs based on a model trained exclusively with the direct ancestors of the selection candidates when compared to a model trained with all individuals in the prior generations (**Table 3**).

In our experimental setting GS was more effective for selecting full-sib families than individual trees either across all selection candidates or within families (**Figures 2**, **3**; **Supplementary Tables S4**, **S5**). Already at the smallest proportions selected, 60% of the top-ranked families for PY could be genomically selected (**Figure 3**). Comparable results of better GS performance for family selection were observed in other *Eucalyptus* (Resende et al., 2017; Simiqueli et al., 2023) and *Pinus* breeding populations (Rios et al., 2021). Rios et al. (2021), working with both real and simulated *Pinus taeda* L. populations, demonstrated an approximately 40% improvement in accuracy of genomic prediction at the family level compared to the individual level for various traits (lignin, tree stiffness, rust, and stem diameter), highlighting the importance of strategies that integrate both family and individual selection. Simiqueli et al. (2023) showed that genomic data accurately predicted and ranked families by the average genomic breeding value across generations in eucalypts, and that the top-ranked full-sib families contained the majority of the top-ranked individual trees. Our results therefore further support the two-stage genomic selection approach proposed earlier (Grattapaglia, 2022), that capitalizes on the benefits of family selection. This involves the initial selection of families based on average GEBVs, followed by subsequent individual genomic selection within the top-performing families, considerably increasing between and within-family selection intensity while optimizing genotyping costs.

### 4.3 High relatedness and low G × E between training and testing populations are key drivers of the realized predictive ability for volume growth

Our study additionally explored the impact of average relationships and G × E interactions between training and testing populations on the RPAs for volume growth (**Table 5**). Incorporating these factors into our analysis allowed us to better understand the nuances and challenges associated with predicting volume growth accurately. We investigated six scenarios based on training populations composed of three trials from the second generation, each with different average relationships (ranging from 3.4E-05 to 2.0E-04) and G × E interactions (ranging from 0.50 to 0.84) (**Supplementary Table S2**) in relation to a testing in the third generation. Scenarios with the highest genetic relationships and lowest G × E interactions (*i.e.*, highest additive genetic correlation) between training and testing populations, using the largest training sets including the direct ancestor trees, under a ssGBLUP model, showed considerably improved predictions for VOL, reaching an RPA of 0.39 (**Table 5**) approximately 3x higher than the RPAs observed when these training population optimizations were not taken into account (**Table 3**). Conversely, scenarios with lower average genetic relationships between training and testing sets (scenarios 1 and 2), resulted in lower RPAs.

These results represent additional evidences to the now established fact that a high level of genetic relatedness between the training and testing populations is the key driver of genomic predictions (reviewed in Grattapaglia et al., 2018; Isik, 2022). Moreover, early studies in forest trees (*e.g.*, Resende et al., 2012; Beaulieu et al., 2014), had shown that G × E interaction significantly influence the transferability of prediction models. Specifically, these studies revealed higher accuracies within the same site or breeding zone, particularly for complex growth traits such as diameter at breast height and total height. In *Pinus taeda*, Resende et al. (2012) found that prediction models developed for one site in Florida could be accurately applied to another site within the same breeding zone with only a marginal loss in predictability (≤ 0.08). However, when these models were applied to sites in different breeding zones, such as the Upper Coastal Plain versus Piedmont, there was a significant decline in accuracy. This emphasizes that G × E severely impacts model transferability across breeding zones. In white spruce, Beaulieu et al. (2014) revealed that the prediction accuracy into untested environments was relatively low for growth traits (with correlations of ≥ 0.24) but was moderately high for wood traits (with correlations of ≥ 0.61), emphasizing the need to phenotype trees in all test environments and model G × E interactions for growth traits.

G × E interaction is a common challenge in tree breeding programs, varying by species, environmental conditions, and the type of planting material used (families or clones) (Grattapaglia, 2014). Correctly ranking individual trees by their GEBVs is crucial for successful GS implementation. Our results indicate that accurately ranking individual trees by their GEBVs could be influenced by environmental factors, and complex gene-environment interactions (Cappa et al., unpublished). Multi-environment GS models improved the predictive ability for across-environment predictions, meaning they could effectively predict the performance of genotypes evaluated in certain environments but not others, which is particularly important for most forest breeding programs. Future research is warranted to explore incorporating environmental data to account for these G × E interactions (Ratcliffe et al., 2019), and investigating gene-environment interactions. By refining training populations and leveraging advanced genomic models like ssGBLUP, tree breeding programs can improve the selection of trees with superior growth traits, ultimately optimizing breeding efficiency and outcomes.

## 5 Conclusions

The primary challenge of experimental research toward the application of GS in forest trees has been the extended time required to complete intergenerational studies by measuring the ultimate phenotypes at harvest age and match them to the predicted genomic values estimated at the seedling stage based on training data from ancestral generations. Few studies to date have therefore been able to experimentally validate genomically selected candidates across tree breeding generations. This crucial step precedes the necessary validation of the GS approach toward its integration into operational tree breeding practice. We expect, however, that more studies will be published in the next years providing increasing evidences in support of this breeding approach that is on its way to transform tree breeding (Grattapaglia, 2022). In this work we contributed experimental data to this topic by comparing the genomic predictions of breeding values (GEBVs) of 825 young seedling candidates for volume growth (VOL), wood density (WD), and pulp yield (PY) to their corresponding estimated breeding values (EBVs) across four-generations in a *Eucalyptus grandis* population. The training population, spanning three generations, assessed the impact of including all genotyped trees (1,918) or exclusively the direct ancestors (67), targeting selection of individuals or families. As expected, given the higher heritabilities of family means when compared to individual tree level, GS was more effective to rank and select families than individual trees. This outcome provides supporting data to a two-stage GS approach to increase selection intensity by screening more families and optimizing the genotyping investment for individual selection within the top-ranked families.

In our experimental settings the performance of GS across generations was quite different depending on the trait under consideration. High RPAs in the range of 0.5 to 0.7 were estimated for WD and PY and satisfactory proportions of top-ranked individual trees and families ranked by EBVs were correctly identified by genomic data. Given the late expression of these traits and the high measurement cost involved, particularly of PY, this result corroborates the potential of adopting GS for these traits at the seedling stage in this *Eucalyptus* breeding population. Interestingly, GS was shown to be slightly more effective as a culling tool of the worst performing individuals, than as a way to identify the winners, a feature that might prove useful in breeding operations to optimize the effort devoted to field testing families or individuals in clonal trials, following the genomic selection step. GS however was not as effective as phenotypic selection for predicting volume growth, providing RPA values below 0.2 in a standard setting with no improvement when only ancestors were used for training. However, when the relationship and population size were maximized and G × E interaction minimized between training and testing generations the RPAs improved to an acceptable value of 0.39. Our result for volume growth contrasts with those of the few other intergenerational studies in forest trees, where realized predictive abilities above 0.7-0.8 were reported. Differences in the effective population size, experimental precision, trait heritability, levels of relatedness, G × E and G × Year interaction between training and testing populations all come into play to define the final outcome of GS, highlighting the importance of experimental studies spanning multiple generations for assessing the expected GS performance in the specific breeding program and environmental context where GS is to be applied.

Finally, another important result of this work was to show that a smaller training population including only direct ancestors of the selection candidates improved genomic predictions, likely by enhancing its level of genetic relationship to the selection candidates. This was the case for wood quality traits but not for volume growth. While this result might indicate that potentially smaller and highly related training populations to the selection candidates could be used in GS, more studies seem necessary to recommend this approach. In any case, the decisive role of relationship in enhancing the performance of GS was further demonstrated in our work when different scenarios varying the level of relationship between training and testing sets were examined. Our study also touched on the impact of G × E interaction as an important determinant of the performance of GS. G × E is a fact of life that will be omnipresent in any tree breeding practice, irrespective of the use of GS or any new genomic technology. High relatedness and low G × E between training and testing populations are indispensable requirements for the successful implementation of GS in forest tree breeding. A corollary derived from these two driving forces is thus that GS implementation will be breeding-population specific, requiring that each organization be creative to develop and validate predictive models for its reference genetic base, environmental settings, target traits, and overall breeding objectives (Grattapaglia, 2022).

## Data Availability

The raw data supporting the conclusions of this article will be made available by the authors, without undue reservation. Requests to access these datasets should be directed to Carmelo Centurión: carmelo.centurion@upm.com.
