# Supplementary material for: Genomic selection in forest trees comes to life: unraveling its potential in an advanced four-generation Eucalyptus grandis population
Source: Front Plant Sci. 2024 Oct 30;15:1462285. doi: 10.3389/fpls.2024.1462285 (PMC11558521; doi:10.3389/fpls.2024.1462285)

Supplementary material

Table S1. Generation, test type (OP = open-pollinated, CP = control-pollinated), number of families and experimental design data for each of the 13 progeny trials from the four-generation breeding program of *E. grandis*.

**Trial number**	**1**	**2**	**3**	**4**	**5**	**6**	**7**	**8**	**9**	**10**	**11**	**12**	**13**
**Generation**	1	1	1	2	2	2	2	3	3	3	3	3	4
**Test Type**	OP	OP	OP	OP	OP	OP	OP	OP	OP	CP	CP	CP	CP
**Number of families**	309	311	60	35	33	31	548	125	125	30	38	29	54
**Number of replicates**	8	8	8	32	32	32	20	8	8	5	5	5	5
**Number of incomplete blocks**	16	16	9	8	8	8	25	10	10	6	6	6	6
**Spacing (m × m)**	4 × 2.25	4 × 2.25	3.5 × 2	3 × 2	3 × 2	3 × 2	3 × 2	3 × 2	3 × 2	3 × 2.4	3 × 2.4	3.5 × 2	3.5 × 2
**Number of tree per plot**	4	4	4	1	1	1	1	4	4	4	4	4	4

Table S2. Average theoretical relationship (lower triangle) and estimated additive genetic correlations (upper triangle) between sites in the first three generations of the *Eucalyptus grandis* population studied. The data include the generation number, trial number, the number of trees with volume growth phenotypes and pedigree records, and the total number of genotyped trees. Bold numbers indicate pairs of training-to-testing sites studied in detail to assess the effect of the average relationships and genotype by environment (G × E) interaction (i.e., additive genetic correlation).

**Generation**	**1**	**1**	**1**	**2**	**2**	**2**	**2**	**3**	**3**	**3**	**3**	**3**
**Trial number**	**1**	**2**	**3**	**4**	**5**	**6**	**7**	**8**	**9**	**10**	**11**	**12**
**No. records**	**7433**	**8092**	**1792**	**829**	**567**	**711**	**6964**	**3158**	**3222**	**576**	**542**	**575**
**Total of genotyped trees**	**0**	**71**	**1**	**40**	**0**	**4**	**773**	**549**	**480**	**0**	**0**	**0**
**1**		0.86	0.55	0.35	0.47	0.53	0.42	0.84	0.53	0.95	0.96	0.95
**2**	8.2E-04		0.17	-0.08	0.03	0.16	-0.02	**0.50**	0.13	0.79	0.86	0.79
**3**	0.0E+00	0.0E+00		0.96	0.97	1.00	0.97	0.91	0.99	0.73	0.64	0.73
**4**	6.0E-04	6.4E-04	5.0E-04		0.99	0.97	0.99	**0.80**	0.98	0.55	0.43	0.54
**5**	6.0E-04	6.3E-04	4.5E-04	5.9E-02		0.97	1.00	0.87	0.99	0.63	0.52	0.63
**6**	6.3E-04	6.3E-04	5.9E-04	6.0E-05	0.0E+00		0.97	0.89	0.99	0.72	0.63	0.72
**7**	1.7E-04	1.8E-04	8.8E-06	1.3E-04	1.4E-04	2.1E-04		**0.84**	0.99	0.60	0.49	0.60
**8**	8.3E-05	**9.1E-05**	1.8E-04	**3.4E-05**	2.6E-05	3.8E-04	**2.0E-04**		0.90	0.92	0.86	0.92
**9**	8.3E-05	9.1E-05	1.8E-04	3.3E-05	2.5E-05	4.1E-04	2.0E-04	2.0E-03		0.71	0.61	0.70
**10**	2.5E-04	2.9E-04	4.5E-05	1.6E-03	1.2E-03	1.0E-02	5.1E-04	1.3E-03	1.3E-03		0.99	1.00
**11**	2.5E-04	3.0E-04	4.8E-05	1.6E-03	1.3E-03	1.1E-02	5.1E-04	1.3E-03	1.3E-03	6.2E-02		0.99
**12**	2.4E-04	2.9E-04	4.3E-05	1.6E-03	1.2E-03	9.5E-03	5.2E-04	1.5E-03	1.4E-03	6.2E-02	6.2E-02	

Table S3. Average and ranges of observed breeding values (EBVs) for each trait and for the entire population of selection candidates, across different genomic selection targets and selection criteria. Within each trait, common letters are not significantly different (α = 0.05) according to the Tukey test. Abbreviations used for traits, genomic selection targets, and selection criteria are detailed in the in the text.

Genomic selection target	Genomic Selection approach	VOL	WD	PY
**All selection candidates**		0.73 (0.08 – 1.66)	0.32 (-1.07 – 1.88)	0.18 (-1.09 – 1.70)
**Individual-tree GS**	**11% top-ranked (*n* = 90)**	0.72^{bc} (0.24 – 1.27)	0.67^{b} (-0.63 – 1.88)	0.82^{b} (-0.19 – 1.56)
	**11% bottom-ranked (*n* = 90)**	0.67^{ab} (0.19 – 1.06)	-0.30^{a} (-1.07 – 1.20)	-0.54^{a} (-1.09 – 0.27)
	**Multiple-trait index selected**	0.61^{a} (0.24 – 1.24)	0.74^{b} (-0.63 – 1.88)	0.69^{b} (-0.46 – 1.48)
	**Equal size random sample**	0.76^{c} (0.28 – 1.24)	0.34^{c} (-1.07 – 1.41)	0.23^{c} (-0.84 – 1.70)
**Individual-tree within-family GS**	**11% top-ranked (*n* = 90)**	0.74^{bc} (0.24 – 1.24)	0.43^{c} (-0.63 – 1.81)	0.70^{d} (-0.46 – 1.60)
	**11% bottom-ranked (*n* = 90)**	0.68^{ab} (0.19 – 1.10)	-0.17^{a} (-1.07 – 0.50)	-0.51^{a} (-1.09 – 0.27)
	**Multiple-trait index selected**	0.65^{a} (0.25 – 1.24)	0.62^{b} (-0.63 – 1.81)	0.51^{b} (-0.46 – 1.60)
	**Equal size random sample**	0.76^{c} (0.08 – 1.25)	0.29^{c} (-0.55 – 1.20)	-0.24^{c} (-0.84 – 0.83)
**Between family GS**	**19% top-ranked (*n* = 10)**	0.74^{a} (0.49 – 0.96)	0.43^{ab} (-0.21 – 1.66)	0.69^{b} (-0.15 – 1.19)
	**19% bottom-ranked (*n* = 10)**	0.68^{a} (0.43 – 0.97)	-0.14^{a} (-0.79 – 0.41)	-0.51^{a} (-0.76 – -0.30)
	**Multiple-trait index selected**	0.64^{a} (0.39 – 0.96)	0.63^{b} (-0.09 – 1.66)	0.50^{b} (-0.28 – 1.19)
	**Equal size random sample**	0.65^{a} (0.36 – 0.92)	0.61^{b} (-0.06 – 1.66)	0.29^{b} (-0.62 – 1.19)

Table S4. Percent correspondence (and absolute numbers in parenthesis) between the number of trees or families that would be genomically ranked at the top and therefore selected at the seedling stage by ssGBLUP (GEBV) and phenotypically selected (EBVs) at age three years for increasing selected proportions within each genomic selection target. Abbreviations used for traits and genomic selection targets are detailed in the text.

Genomic selection target	Selected proportion (%)	No. families	No. trees	VOL	WD	PY
**Individual-tree GS**	5	-	40	2.5 (1)	27.5 (11)	35.0 (14)
	11	-	90	5.6 (5)	35.6 (32)	41.1 (37)
	15	-	125	16.8 (21)	28.8 (36)	48.0 (60)
	20	-	165	22.4 (37)	30.9 (51)	49.7 (82)
	25	-	210	31.0 (65)	33.8 (71)	49.0 (103)
**Individual-tree within-family GS**	5	5	45	0.0 (0)	21.4 (9)	31.1 (14)
	11	10	90	9.2 (8)	10.3 (9)	32.2 (28)
	16	15	135	26.5 (35)	13.6 (18)	30.3 (40)
	22	20	180	25.9 (45)	18.6 (33)	32.8 (58)
	27	25	225	32.0 (70)	32.6 (72)	38.7 (86)
**Between family GS**	9	5	-	0.0 (0)	40.0 (2)	60.0 (3)
	19	10	-	20.0 (2)	20.0 (2)	60.0 (6)
	28	15	-	40.0 (6)	26.7 (4)	53.3 (8)
	37	20	-	40.0 (8)	40.0 (8)	60.0 (12)
	46	25	-	48.0 (12)	56.0 (14)	72.0 (18)

Table S5. Percent correspondence (and absolute numbers in parenthesis) between the number of trees or families that would be genomically ranked at the bottom and therefore excluded at the seedling stage by ssGBLUP (GEBV) and phenotypically selected (EBVs) at age three years for increasing selected proportions within each genomic selection target. Abbreviations used for traits and genomic selection targets are detailed in the text.

Genomic selection target	Selected proportion (%)	No. families	No. trees	VOL	WD	PY
**Individual-tree GS**	5	-	40	7.5 (3)	35.0 (14)	35.0 (14)
	11	-	90	15.6 (14)	51.1 (46)	52.2 (47)
	15	-	125	22.4 (28)	44.0 (55)	57.6 (72)
	20	-	165	25.5 (42)	47.9 (79)	63.6 (105)
	25	-	210	29.1 (61)	51.0 (107)	81.4 (171)
**Individual-tree within-family GS**	5	5	45	19.9 (7)	44.4 (20)	47.7 (21)
	11	10	90	19.1 (17)	27.8 (25)	37.1 (33)
	16	15	135	27.6 (37)	18.9 (25)	52.2 (70)
	22	20	180	30.7 (55)	34.5 (61)	60.3 (108)
	27	25	225	33.0 (74)	33.3 (74)	58.4 (129)
**Between family GS**	9	5	-	20.0 (1)	60.0 (3)	60.0 (3)
	19	10	-	30.0 (3)	50.0 (5)	50.0 (5)
	28	15	-	40.0 (6)	33.3 (5)	73.3 (11)
	37	20	-	45.0 (9)	55.0 (11)	85.0 (17)
	46	25	-	52.0 (13)	56.0 (14)	84.0 (21)

Figure S1. Network representation of pedigree-based relationship matrix for the 2,971 genotyped trees from the four-generation *Eucalyptus grandis* breeding population. The colored circles represent the half-sib and full-sib *Eucalyptus grandis* families, whereas larger clusters and edges (black) represent connections among these families. The network connects trees that have theoretical relationship > 0.001. The colors represent the four generations: first-generation (violet), second-generation (green), third-generation (yellow), and fourth-generation (blue). The central cluster comprises 67 direct ancestors along with relatives as aunts (0.125) and great-aunt (0.0625) spanning three generations (violet, green, and yellow dots). These trees share genetic relationships with the total of 1,053 genotyped trees, which are fourth-generation selection candidates (blue dots). On the other hand, the outer ring cluster shows more distant genetic connections (< 0.001) with the selection candidates, including fewer than 1,851 trees given that excluding, for example, aunts and great-aunts of the selected trees within the fourth generation.

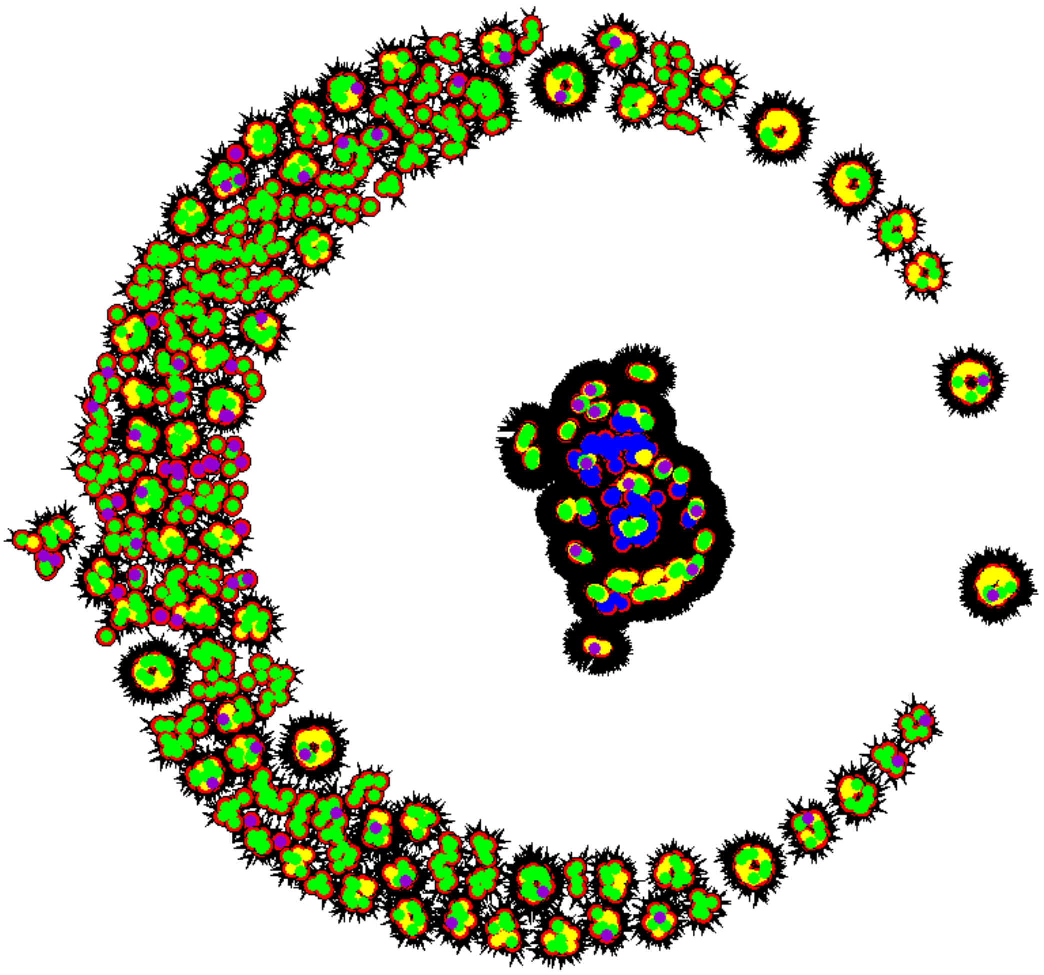

Figure S2. Density plots showing the distribution of the average theoretical relationship for the 825 trees in the testing population with the 67 genotyped direct ancestors and all the 1,918 genotyped trees from the training population.

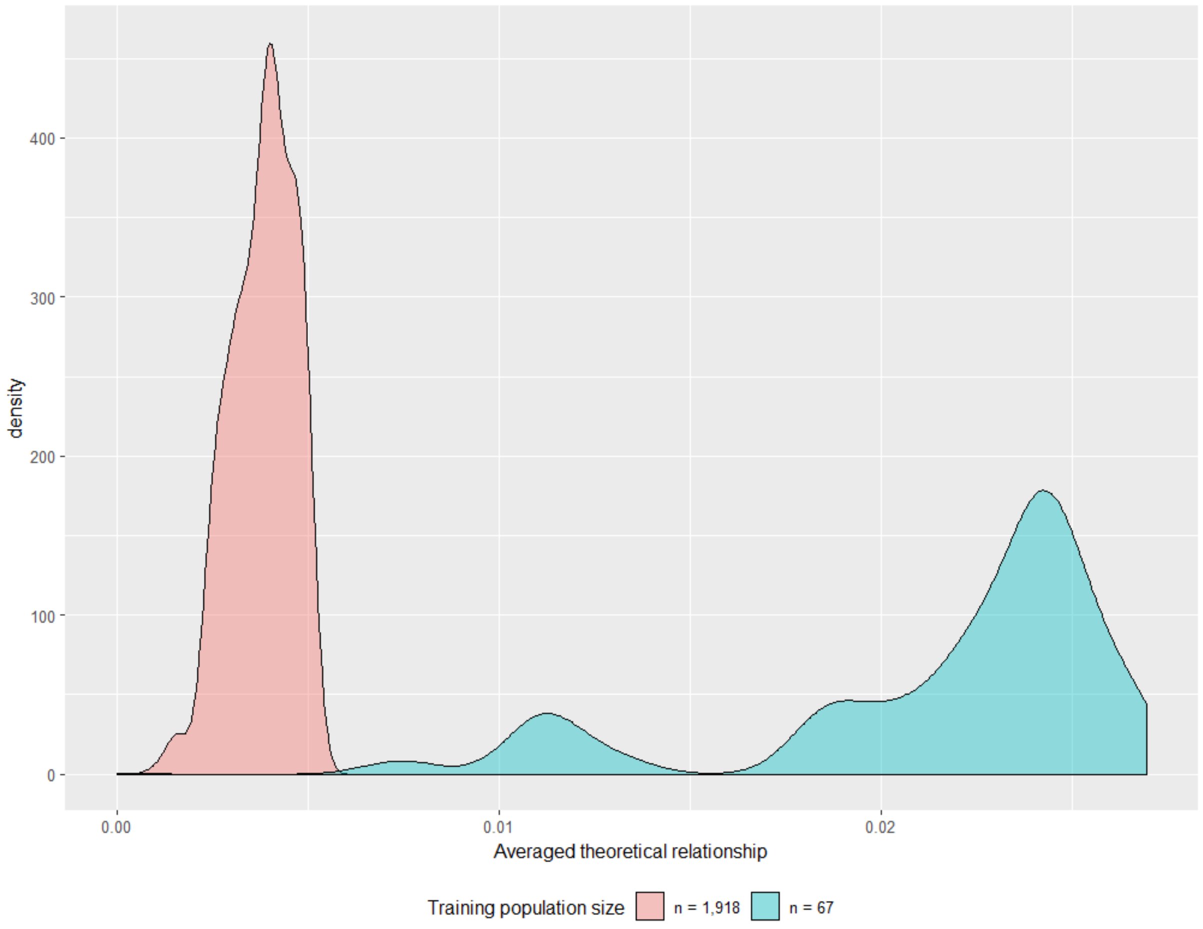

Supplement: Supplementary file 1 [file DataSheet1.pdf]
